# Elucidating the clinical and genetic spectrum of inositol polyphosphate phosphatase *INPP4A*-related neurodevelopmental disorder

**DOI:** 10.1016/j.gim.2024.101278

**Published:** 2024-09-21

**Authors:** Lettie E. Rawlins, Reza Maroofian, Stuart J. Cannon, Muhannad Daana, Mina Zamani, Shamsul Ghani, Joseph S. Leslie, Nishanka Ubeyratna, Nasar Khan, Hamid Khan, Annarita Scardamaglia, Robin Cloarec, Shujaat Ali Khan, Muhammad Umair, Saeid Sadeghian, Hamid Galehdari, Almundher Al-Maawali, Adila Al-Kindi, Reza Azizimalamiri, Gholamreza Shariati, Faraz Ahmad, Amna Al-Futaisi, Pedro M. Rodriguez Cruz, Ainara Salazar-Villacorta, Moustapha Ndiaye, Amadou G. Diop, Alireza Sedaghat, Alihossein Saberi, Mohammad Hamid, Maha S. Zaki, Barbara Vona, Daniel Owrang, Abdullah M. Alhashem, Makram Obeid, Amjad Khan, Ahmad Beydoun, Marwan Najjar, Homa Tajsharghi, Giovanni Zifarelli, Peter Bauer, Wejdan S. Hakami, Amal M. Al Hashem, Rose-Mary N. Boustany, Lydie Burglen, Shahryar Alavi, Adam C. Gunning, Martina Owens, Ehsan G. Karimiani, Joseph G. Gleeson, Mathieu Milh, Somaya Salah, Jahangir Khan, Volker Haucke, Caroline F. Wright, Lucy McGavin, Orly Elpeleg, Muhammad I. Shabbir, Henry Houlden, Michael Ebner, Emma L. Baple, Andrew H. Crosby

**Affiliations:** 1 Department of Clinical and Biomedical Sciences (Medical School), Faculty of Health and Life Sciences, University of Exeter, Royal Devon and Exeter Hospital, Exeter, United Kingdom; 2 Peninsula Clinical Genetics Service, Royal Devon University Healthcare NHS Foundation Trust, Exeter, United Kingdom; 3 Department of Neuromuscular Diseases, Queen Square, Institute of Neurology, University College London, London, United Kingdom; 4 Child Development Center, Clalit Health Care Services, Jerusalem, Israel; 5 Department of Biology, Faculty of Sciences, Shahid Chamran University of Ahvaz, Ahvaz, Iran; 6 Narges Medical Genetics and Prenatal Diagnosis Laboratory, Ahvaz, Iran; 7 Department of Biological Sciences, International Islamic University Islamabad, Islamabad, Pakistan; 8 Inter-disciplinary Nanoscience Center, Aarhus University, Aarhus, Denmark; 9 Centre de Référence Déficiences Intellectuelles et Polyhandicaps de causes rares, APHM, Hõpital de la Timone-Enfants, Marseille, France; 10 Department of Life Sciences, School of Science, University of Management and Technology, Lahore, Pakistan; 11 Medical Genomics Research Department, King Abdullah International Medical Research Center, King Saud Bin Abdulaziz University for Health Sciences, King Abdulaziz Medical City, Ministry of National Guard Health Affairs, Riyadh, Saudi Arabia; 12 Department of Pediatric Neurology, Golestan Medical, Educational, and Research Center, Ahvaz Jundishapur University of Medical Sciences, Ahvaz, Iran; 13 Department of Genetics, College of Medicine and Health Sciences, Sultan Qaboos University, Muscat, Oman; 14 Genetic and Developmental Medicine Clinic, Department of Genetics, Sultan Qaboos University Hospital, Muscat, Oman; 15 Department of Medical Genetics, Faculty of Medicine, Ahvaz Jundishapur University of Medical Sciences, Ahvaz, Iran; 16 Department of Child Health, College of Medicine and Health Sciences, Sultan Qaboos University, Muscat, Oman; 17 Centro Nacional de Análisis Genómico, Barcelona, Spain; 18 Department of Neurology, Centre Hospitalier National Universitaire de Fann, Dakar, Senegal; 19 Diabetes Research Center, Health Research Institute, Ahvaz Jundishapur University of Medical Sciences, Ahvaz, Iran; 20 Department of Molecular Medicine, Biotechnology Research Center, Pasteur Institute of Iran, Tehran, Iran; 21 Department of Clinical Genetics, Human Genetics and Genome Research Institute, National Research Centre, Cairo, Egypt; 22 Institute of Human Genetics, University Medical Center Göttingen, Göttingen, Germany; 23 Institute for Auditory Neuroscience and InnerEarLab, University Medical Center Göttingen, Göttingen, Germany; 24 Department of Radiology, Prince Sultan Medical Military City, Riyadh, Saudi Arabia; 25 The American University of Beirut, Beirut, Lebanon; 26 Faculty of Biological Sciences, Department of Zoology, University of Lakki Marwat, Lakki, Khyber Pakhtunkhwa, Pakistan; 27 Institute for Medical Genetics and Applied Genomics, University of Tübingen, Tübingen, Germany; 28 School of Health Sciences, Division of Biomedicine, University of Skovde, Skovde, Sweden; 29 Centogene GmbH, Rostock, Germany; 30 Department of Pediatrics, Prince Sultan Military Medical City, Riyadh, Saudi Arabia; 31 Developmental Brain Disorders Laboratory, Imagine Institute, INSERM UMR 1163, Paris, France; 32 Centre de Référence des Malformations et Maladies congénitales du cervelet et Département de génétique, Hõpital Trousseau, APHP, Sorbonne Université, Paris, France; 33 Palindrome, Isfahan, Iran; 34 Exeter Genomics Laboratory (NHS South West Genomic Laboratory Hub), Royal Devon University Healthcare NHS Foundation Trust, Exeter, United Kingdom; 35 Molecular and Clinical Sciences Institute, St. George’s, University of London, London, United Kingdom; 36 Department of Medical Genetics, Next Generation Genetic Polyclinic, Mashhad, Iran; 37 Department of Neurosciences, University of California San Diego, La Jolla, CA; 38 Rady Children’s Institute for Genomic Medicine, San Diego, CA; 39 Aix-Marseille Université, APHM, Service de Neurologie Pédiatrique, Hõpital de la Timone-Enfants, Marseille, France; 40 Department of Genetics, Hebrew University Medical Center, Hadassah, Jerusalem, Israel; 41 Leibniz-Forschungsinstitut für Molekulare Pharmakologie (FMP), Berlin, Germany; 42 Department of Biology, Chemistry, Pharmacy, Freie Universität Berlin, Berlin, Germany; 43 University Hospitals Plymouth NHS Trust, Plymouth, United Kingdom; 44 University of Plymouth, Plymouth, United Kingdom

**Keywords:** Endocytosis, INPP4A, Neurodevelopmental disorder, Phosphoinositide phosphatase, Translation reinitiation

## Abstract

**Purpose:**

Biallelic *INPP4A* variants have recently been associated with severe neuro-developmental disease in single-case reports. Here, we expand and elucidate the clinical-genetic spectrum and provide a pathomechanistic explanation for genotype-phenotype correlations.

**Methods:**

Clinical and genomic investigations of 30 individuals were undertaken alongside molecular and in silico modelling and translation reinitiation studies.

**Results:**

We characterize a clinically variable disorder with cardinal features, including global developmental delay, severe-profound intellectual disability, microcephaly, limb weakness, cerebellar signs, and short stature. A more severe presentation associated with biallelic *INPP4A* variants downstream of exon 4 has additional features of (ponto)cerebellar hypoplasia, reduced cerebral volume, peripheral spasticity, contractures, intractable seizures, and cortical visual impairment. Our studies identify the likely pathomechanism of this genotype-phenotype correlation entailing translational reinitiation in exon 4 resulting in an N-terminal truncated INPP4A protein retaining partial functionality, associated with less severe disease. We also identified identical reinitiation site conservation in *Inpp4a*^−/−^ mouse models displaying similar genotype-phenotype correlation. Additionally, we show fibroblasts from a single affected individual exhibit disrupted endocytic trafficking pathways, indicating the potential biological basis of the condition.

**Conclusion:**

Our studies comprehensively characterize *INPP4A*-related neurodevelopmental disorder and suggest genotype-specific clinical assessment guidelines. We propose that the potential mechanistic basis of observed genotype-phenotype correlations entails exon 4 translation reinitiation.

## Introduction

Phosphatidylinositol (PtdIns) is a fundamental membrane phospholipid that can be reversibly phosphorylated at the 3, 4, and 5 positions of the inositol ring to produce phosphoinositides (PIs) at membrane bilayers.^1^ PIs are important for numerous cellular processes, including cytoskeleton remodeling, intracellular membrane trafficking, signal transduction, and control of cell growth and survival.^1,2^ The spatial and temporal expression of PIs is tightly regulated by specific phosphoinositide kinases and phosphatases.^3^ Pathogenic variants within genes encoding phosphoinositide phosphatases have previously been associated with inherited neurodevelopmental disorders, including OCRL inositol polyphosphate-5-phosphatase (*OCRL*, Lowe syndrome, OMIM #309000),^4^ inositol polyphosphate-5-phosphatase E (*INPP5E*, Joubert syndrome 1/MORM (mental retardation, truncal obesity, congenital nonprogressive retinal dystrophy, and micropenis), OMIM #213300/#610156),^5,6^ inositol polyphosphate-5-phosphatase K (*INPP5K*, congenital muscular dystrophy with intellectual disability and cataracts, OMIM #617404),^7^ and synaptojanin 1 (SYNJ1, developmental and epileptic encephalopathy 53, OMIM #617389).^8^ Neurodevelopmental disorders have also been associated with pathogenic variants in phosphoinositide kinases, including phosphatidylinositol-4-kinase alpha (*PI4KA*, polymicrogyria with cerebellar hypoplasia and arthrogryposis/ autosomal recessive spastic paraplegia 8, OMIM #616531/#619621),^9^ phosphatidylinositol-4-kinase type 2 alpha (PI4K2A, neurodevelopmental disorder with hyperkinetic movements, seizures and structural brain abnormalities, OMIM #620732),^10^ and phosphatidylinositol-3-kinase class 2 alpha (*PIK3C2A*, oculoskeletodental syndrome OMIM #618440).^11^

Inositol polyphosphate-4-phosphatase type IA (INPP4A, HGNC:6074) is an evolutionarily conserved phosphoino sitide phosphatase that dephosphorylates phosphatidyl inositol- 3,4-bis-phosphate (PtdIns(3,4)P_2_) to phosphatidyl inositol-3-phosphate (PtdIns(3)P) at the 4 position of the inositol ring.^12^ PtdIns(3,4)P_2_ plays an important role in clathrin-mediated endocytosis^13,14^ and is an important membrane bound signaling molecule within the phosphatidylinositol-3-kinase (PI3K) pathway, which activates protein kinase B (serine/threonine kinase 1, AKT) via 3-phosphoinositide-dependent kinase and mammalian target of rapamycin complex 2 (Supplemental Figure 1A and B).^15,16^ Thus dephosphorylation of PtdIns(3,4)P_2_ by INPP4A acts as a negative downstream regulator of PI3K-AKT signaling. INPP4A plays a role in the nucleus to regulate cell cycle progression, proliferation, and apoptosis^17^ and has been shown to act as a suppressor of glutamate excitotoxicity through regulation of neuronal apoptosis within the central nervous system.^18,19^ INPP4A has 6 protein coding transcripts,^20^ with several, including NM_001351425.2 being highly expressed in the brain, particularly within the cerebellum.^21^

Previous publications have described single families with biallelic *INPP4A* loss of function (LoF)^22-24^ and candidate missense or protein altering variants^25-28^ as the cause of disease in individuals affected by a variable neurodevelopmental disorder, alongside mostly limited clinical information for affected individuals with a largely inconsistent phenotype. Here, we present our genetic and clinical findings in 10 new families (14 affected individuals) and detail our review of previously published families harboring candidate pathogenic *INPP4A* variants to more precisely characterize *INPP4A*-related neurodevelopmental disorder.

## Materials and Methods

Research was performed with informed consent from the study participants or their legal guardians, according to institutional and international guidelines for studies with human participants and materials. Affected individuals were identified by their clinician and using GeneMatcher (Baylor-Hopkins Center for Mendelian Genomics, https://genematcher.org).^29^ Phenotypic information was obtained by the clinical care provider using a targeted questionnaire. DNA was extracted from blood/buccal samples before exome sequencing and variant calling using standard techniques. Variants failing quality filters or present at a frequency of >0.1% or with >1 homozygous individual in gnomAD (v.2.1.1 or v.3.1.2)^30^ or in our in-house database were excluded. Potentially deleterious biallelic exonic, non-synonymous, and synonymous with predicted splicing impact or intronic at ±6 nucleotides variants were prioritized. Unique primers were used for amplification and bidirectional dideoxy sequencing of all *INPP4A* variants identified. For additional details about exome sequencing and variant filtering methodologies used, see Supplemental Methods.

Protein modeling images were created in PyMOLv2.5.5 (https://pymol.org/2/#page-top) using AlphaFold model of INPP4A (AF-Q96PE3-F1).^31^ Possible downstream noncanonical start sites were identified using an in-house in silico prediction tool (Supplemental Methods).

Studies of Akt/mTORC1 signaling and endocytosis in fibroblasts from an affected individual are detailed in Supplemental Text 1. For transferrin endocytosis assay, cells were loaded with Alexa568-conjugated transferrin, surface bound transferrin was removed by acid wash, and cells were fixed and imaged on a NikonCSU spinning disc microscope. For immunoblot analysis cells were lysed in 1% Igepal buffer, lysates were loaded on sodium dodecylsulfate polyacrylamide gel electrophoresis (SDS PAGE) gels, blotted on nitrocellulose. Antibodies were detected by chemiluminescence. Please refer to Supplemental Methods for further descriptions.

## Results

### Clinical features of *INPP4A*-related neurodevelopmental disorder

**Figure 1:**
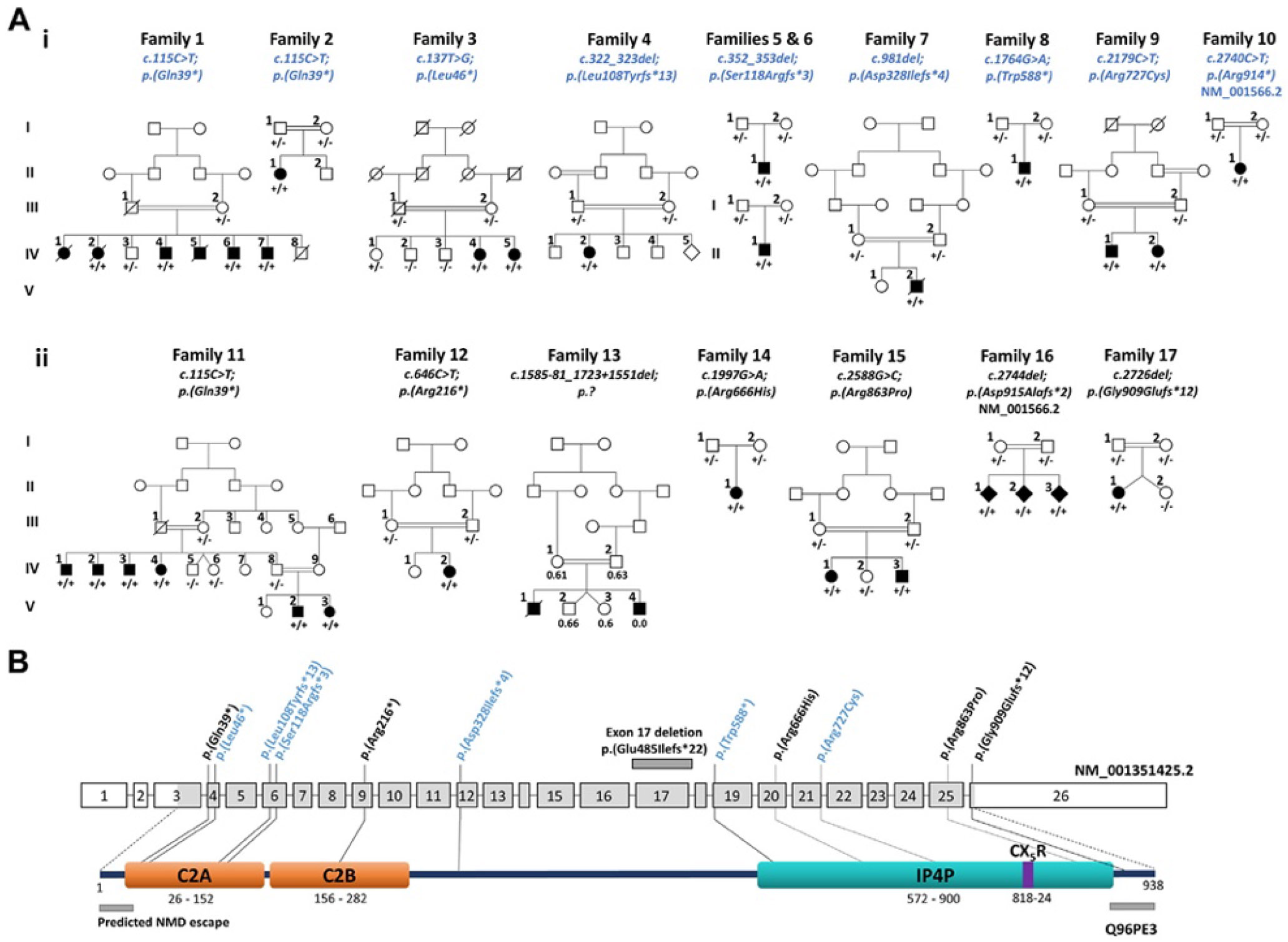
Pedigrees of 17 families with individuals affected with *INPP4A*-related disorder showing the location of biallelic variants within *INPP4A*. Ai. Pedigrees of 10 newly identified families with individuals affected by *INPP4A*-related disorder (black shaded symbols) who are homozygous for pathogenic *INPP4A* variants listed above in blue (+), with co-segregation confirmed in other family members (− indicates wild-type allele). Aii. Pedigrees of 7 previously published families with an additional unpublished individual (V:3, family 11; Banihashemi et al^23^), family 12 (Özkan Kart et al^24^), family 13 (Sheffer et al^22^), family 14 (Olson et al^26^), family 15 (Al-Kasbi et al^27^), family 16 (Najmabadi et al^25^), and family 17 (Hecher et al^28^), with the homozygous *INPP4A* (NM_001351425.2) variant listed above in black. Numbers listed underneath individuals in family 13 indicate the copy number for this variant region. B. *INPP4A* (NM_001351425.2) gene schematic with grey boxes indicating coding regions, unshaded boxes indicating noncoding exons, and the domain structure below (Q96PE3) displaying the N terminus location of two C2 domains (orange) and a C terminus inositol polyphosphate 4-phosphatase (IP4P) domain (teal) with a catalytic CX_2_R motif region (purple) that mediates phosphatase activity. The location of *INPP4A* variants reported in the present study are shown in blue and previously published variants in black. A 1770-bp deletion (NC_000002.12:g.98555472_ 98557243del)^22^ including the whole of exon 17 is indicated by a dark gray box. Regions predicted to escape NMD are indicated by gray boxes below the protein structure.

Table 1 summarizes the core phenotypic features of autosomal recessive INPP4A-associated neurodevelopmental disorder observed in 21 individuals (including 12 not previously reported) aged 6 months to 40 years from 13 unrelated families (33% female and 67% male) with biallelic likely pathogenic/pathogenic INPP4A variants (families 1, 2, 4-9, 11-13, and 17, Figure 1A, Supplemental Table 1). A further 5 families (9 affected individuals, 3 new) were identified with notable phenotypic overlap and biallelic *INPP4A* variants classified as of uncertain clinical significance (VUS) according to ACMG/ACGS criteria (families 3, 10, 14-16, Figure 1A, Table 1, Supplemental Table 1, shaded gray).^34,35^ A full clinical description for all 30 affected individuals is provided in Supplementary text 2 and Supplemental Table 1 including new phenotypic details for 6 previously published families (Families 11-15, 17 and an additional affected individual, V:3 in family 11).

**Figure 2:**
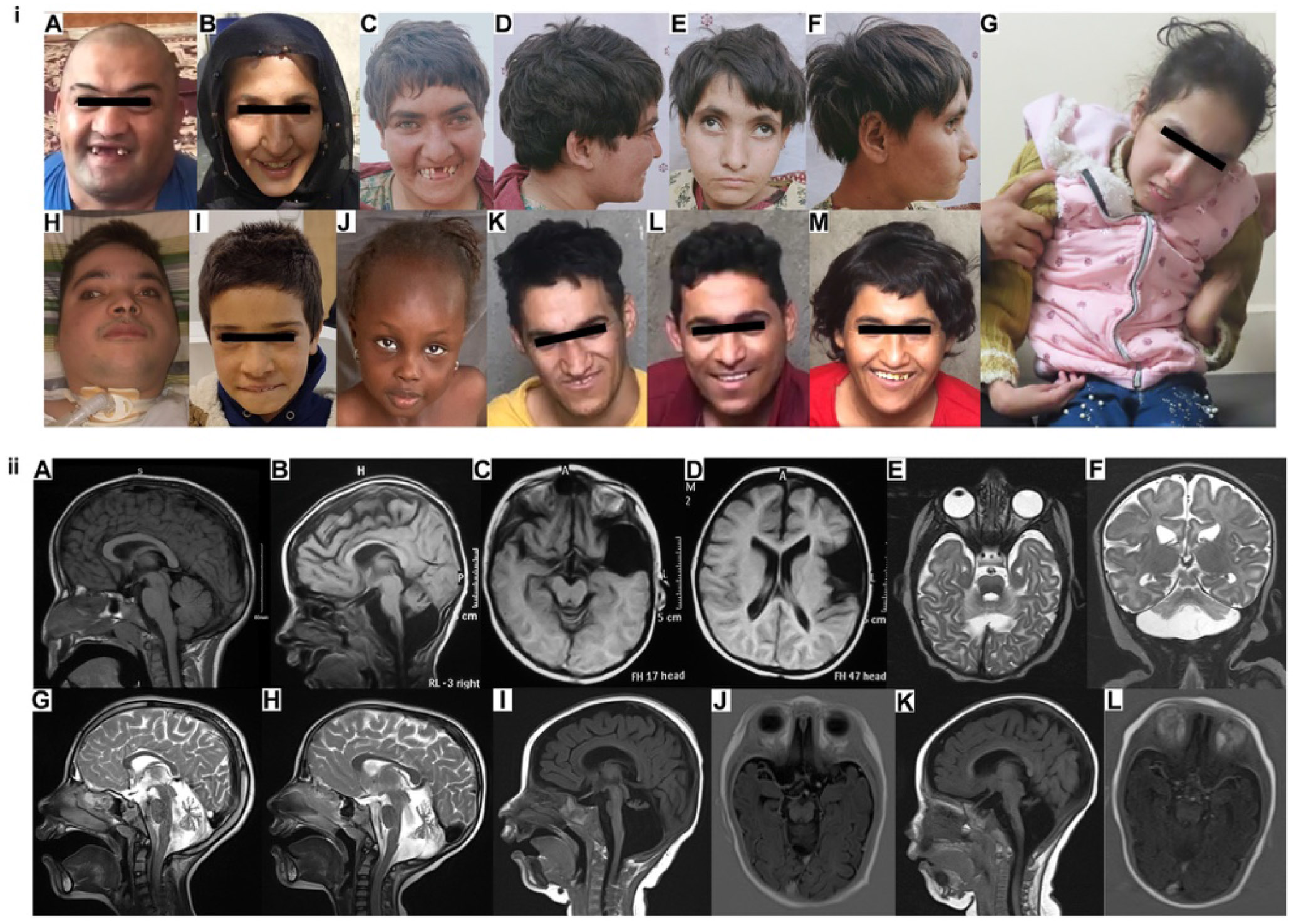
Clinical photographs and MRI neuroimaging of affected individuals with *INPP4A*-related disorder. 2i A-M. Facial photographs of eleven affected individuals with *INPP4A*-related disorder from 8 families ([A] family 1, IV:6, [B] family 2, II:1, [C and D] individual IV:4 and [E and F] IV:5 from family 3, [G] family 4, IV:2, [H] family 5, II:1, [I] family 9, IV:1, [J] family 10, II:1, [K] individual IV:1, [L] individual IV:2, and [M] individual IV:4 from family 11), with characteristic facial features, including a long nose with pointed nasal tip, short philtrum, thin upper lip, thick eyebrows, strabismus, hypertelorism, and dental anomalies, including hypodontia and absent central incisors. G. Upper body image of individual IV:2 from family 4, demonstrating severe spasticity of the upper limbs with fixed flexion at the wrists. 2iiA-L. Neuroimaging of affected individuals with *INPP4A*-related disorder. For all but one of the cases, only single time points are available. Therefore, we have chosen to use the terminology hypoplasia or lack of cerebral/cerebellar volume instead of the term atrophy, which implies progressive loss of brain volume over time. We have used the term ventriculomegaly in preference to ventricular dilatation. A. MRI brain imaging showing sagittal T1 image for individual II:1 from family 2 showing normal appearances. B-D. individual IV:2, family 4 B. sagittal T1 image demonstrating cerebellar vermis hypoplasia, slender brainstem and thin corpus callosum. C and D. Axial T1 images showing an incidental left temporal pole arachnoid cyst and moderate reduction in cerebral volume. E and F. Individual V:2, family 7. E. Axial T2 showing generalized hypomyelination with some myelination present in the posterior pons. F. Coronal T2 demonstrating cerebellar hypoplasia with rudimentary vermis. G. Midline sagittal T2 of individual IV:1 (family 8) showing generalized lack of cerebral volume, pontocerebellar hypoplasia with partially absent vermis, intact corpus callosum, and normal myelination. H. Individual IV:2 from family 8, sagittal T2 showing slender pons with cerebellar vermian hypoplasia. There is normal myelination and corpus callosum volume. I and J. Individual IV:1, family 13, I sagittal T1 showing pontocerebellar hypoplasia with near absent vermis. J. Axial image showing hypomyelination. K and L. Sibling IV:3, family 13, K sagittal T1 showing similar features of pontocerebellar hypoplasia, rudimentary vermis, and generalized lack of volume with intact but slender corpus callosum. L. Axial image showing hypomyelination.

All 30 affected individuals with confirmed or suspected *INPP4A*-related disorder reported to date present in infancy (between birth and 2 years). Affected individuals displayed a consistent pattern of mild craniofacial dysmorphism, involving a long nose with pointed tip and short philtrum, thin upper lip, thick eyebrows, hypertelorism and dental anomalies including hypodontia and absent central incisors (Figure 2iA-M). The most prevalent clinical phenotypes shared among all affected individuals included global developmental delay, severe to profound intellectual disability, and muscle weakness affecting the lower extremities, with variability in the upper extremities. This sometimes manifested with spasticity and/or axial hypotonia (Table 1). In almost all cases, gross motor delay was severe, with 92% (22/24) of affected individuals >1.5 years being nonambulatory. Additionally, 54% (13/24) were able to crawl or walk quadrupedally (see Supplemental Video 1), whereas 46% (11/24) were unable to sit without support. A total of 44% (11/25) of affected individuals were nonverbal and expressive language appeared to be more significantly impaired than receptive language.

In cases where detailed information on neurological findings was available, cerebellar signs were frequently reported, comprising dysarthria, nystagmus, tremor, and ataxia in 81% (13/16) of cases (Supplemental Video 2). Other neurological manifestations included choreoathetoid and/or dystonic movements, myoclonus and seizures (myoclonic, generalized tonic-clonic [GTC], and tonic). Electroencephalogram studies were available for 10 individuals, revealing findings such as interictal generalized polyspike epileptiform activity, generalized slow waves, hypsarrythmia, and burst suppression (see Supplemental Results).

Neuroimaging (magnetic resonance imaging [MRI]/computed tomography) was performed for 77% (20/26) of individuals affected by INPP4A-related disorder (Figure 2iiA-L). The results revealed normal findings in 30% (6/20) (Figure 2iiA), cerebellar hypoplasia in 65% (13/20) (Figure 2iiB-L), additional hindbrain hypoplasia/abnormalities (pontocerebellar hypoplasia) in 40% (8/20), and reduced cerebral volume in 40% (8/20). Other observations included ventriculomegaly in 20% (4/20), thinning of the corpus callosum in 20% (4/20) (Figure 2iiB,I, and K) and hypomyelination in 15% (3/20). In individual II:2 (family 5), a Dandy Walker variant with hyperdense areas in bilateral parietooccipital cortex and subcortical white matter was reported (although neuroimaging was not available for review), and in individual II:2 (family 4), an incidental left temporal pole arachnoid cyst was observed (Figure 2iiC and D).

Ocular and visual abnormalities included strabismus and reduced acuity, with cortical visual impairment observed in some cases. Behavioral features encompassed agitation or irritability, repetitive movements (commonly head shaking, Supplemental Video 3) and impaired social communication. Two individuals also displayed aggressive episodes. Additional variable clinical features included microcephaly (up to −9.77 SDS) and short stature (up to −8.59 SDS).

### Identification of homozygous *INPP4A* variants

Table 2 lists the *INPP4A* variants reported in this study (see Supplemental Table 2 for variant annotation in all transcripts present in National Center for Biotechnology Information). The families newly described here (families 1-10) were identified through collaborative networks and GeneMatcher,^29^ in which exome sequencing studies identified 8 (7 novel, 1 previously published)^23^ rare (absent or low allele frequency in gnomADv2.1.1/v4.1.0,^30^ Table 2) homozygous *INPP4A* (NM_001351425.2) variants. Alongside this, we also reassessed 7 homozygous candidate pathogenic *INPP4A* variants identified by exome sequencing in 7 previously published families (Table 2). Affected individuals from family 1 (current study) are homozygous for the same *INPP4A* NM_001351425.2:c.115C>T p.(Gln39*) variant and are likely ancestrally related to family 11 (Banihashemi et al^23^), given their residence in the same region of Iran. A further affected Pakistani individual (II:1, family 2) was also homozygous for the c.115C>T; p.(Gln39*) variant indicating it entails a regional founder variant. Similarly, Palestinian families 5 and 6, although not known to be related, share the same likely regional founder pathogenic *INPP4A* NM_001351425.2:c.352_353del p.(Ser118Argfs*3) variant.

### Protein truncating *INPP4A* variants

**Figure 3:**
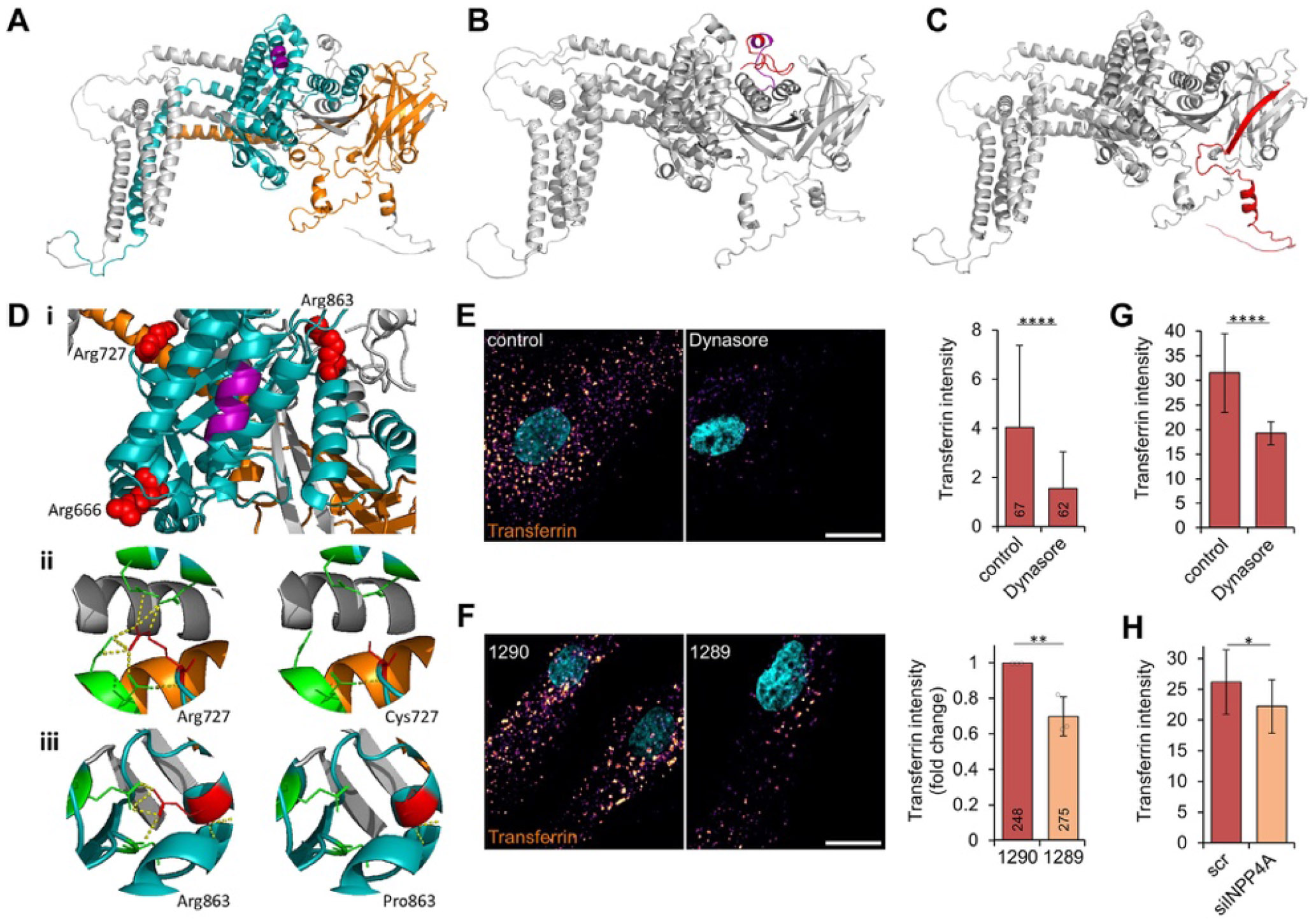
Protein modeling and functional studies confirm *INPP4A* variants result in altered INPP4A protein conformation and impaired endocytosis. A-D. Whole protein models of INPP4A (NM_001351425.2) showing A. functional domains C2A (26-152, orange); C2B (156-282, orange); IP4P (572-900, teal); CX_5_R (818-24, purple). B. Impact of putative NMD escaping variant c.2726del p.(Gly909Glufs*12) with frameshift (909-920, purple) and lost residues due to premature truncation (921-938, red). C. Residues lost because of proposed translation reinitiation at c.163 (1-54, red). D. Protein models of* INPP4A* missense variants. i. Three missense variants (p.(Arg727Cys), p.(Arg666His), and p.(Arg863Pro), red) surrounding the CX_5_R domain (purple). Differences in polar contacts (yellow dashed) between wild-type (left) and mutant (right) proteins for ii. p.(Arg727Cys) and iii. p.(Arg863Pro) in red, respectively. p.(Arg666His) showed no change in polar contacts. E. Dynasore blocks endocytosis in unaffected human fibroblasts. Left panel: Representative images of fibroblasts treated with vehicle control or 80 μM Dynasore, pulsed with CF568-conjugated transferrin, fixed, and stained with DAPI (cyan), scale bar = 20 μm. Right panel: Quantification of transferrin intensity (CF568 integrated density per area), *t* test (*n* = cells) *P* = .0000002, number of analyzed cells indicated in the bar graph. F. Impaired endocytosis in fibroblasts from affected individual IV:2 from family 9 with homozygous *INPP4A* p.(Arg727Cys) variant (1289), compared with heterozygous mother (1290, individual III:1, family 9). Left panel: Representative images of affected INPP4A fibroblasts pulsed with CF568-conjugated transferrin, fixed, and stained with DAPI (cyan), scale bar = 20. Right panel: Quantification of transferrin intensity (CF568 integrated density per area) in *n* = 3 independent experiments compared by one-way ANOVA (*P* = .009), number of analyzed cells indicated in the bar graph. G. Dynasore blocks endocytosis in U2OS cells. Quantification of transferrin intensity (CF568 integrated density per area) in U2OS cells treated with vehicle control or 80 μM Dynasore, t test (*n* = fields of view) *P* = .00000009. H. Impaired endocytosis in INPP4A knockdown cells. Quantification of transferrin intensity (CF568 integrated density per area), *t* test (*n* = 3 independent experiments) *P* = .042, in U2OS cells transfected with scrambled control (scr) or INPP4A-directed siRNA. All bar graphs show mean ± stdev. * *P* < .05, ** *P* < .01, *** *P* < .001, **** *P* < .0001.

Five frameshift *INPP4A* variants were identified, including 3 pathogenic predicted LoF frameshift *INPP4A* variants (families 4-7) likely to undergo mRNA degradation by nonsense-mediated decay (NMD) (Figure 1B, Table 2). The likely pathogenic *INPP4A* NM_001351425.2:c.2726del p.(Gly909Glufs*12) variant identified in family 17^28^ is located in the final coding exon and therefore expected to escape NMD, encoding an INPP4A protein with 11 altered amino acids followed by a stop codon leading to premature truncation of 19 amino acids from the C terminus. Protein modeling studies of this variant suggest a minor misfolding of protein structure (Figure 3A and B). The c.2740C>T p.(Arg914*) and c.2744del p.(Asp915Alafs*2) variants (Families 10 and 15)^25^ affect only 1 of the 6 identified INPP4A protein coding transcripts (NM_001566.2, Supplemental Figure 2B) and are classified as VUS according to ACMG/ACGS variant classification criteria (Table 2). Four nonsense *INPP4A* variants were identified (families 1-3, 8, 11, and 12, Table 2). The c.646C>T p.(Arg216*) and c.1764G>A p.(Trp588*) variants are predicted to undergo NMD, although c.115C>T p.(Gln39*) and c.137T>G p.(Leu46*) variants, located toward the N terminus, were identified to have likely downstream in-frame noncanonical reinitiation sites (Supplemental Table 3).

### Downstream translation reinitiation sites associated with p.(Gln39*) and p.(Leu46*) variants

After the introduction of premature termination codons in exon 4 by c.115C>T p.(Gln39*) and c.137T>G p.(Leu46*) variants, reinitiation of translation from the next potential inframe start codon with moderate Kozak strength (CTG at c.163 in exon 5) would generate a polypeptide lacking the N-terminal 54 amino acids of INPP4A, comprising <6% of the protein and entailing mostly low-confidence unstructured residues of the first beta-strand of the C2A domain (Figure 3C). CTG is the most widely used start site after ATG, and the start codon at c.163 is the only CTG of the noncanonical sites identified.

### Missense *INPP4A* variants

A further 3 rare homozygous missense variants were identified (novel variant NM_001351425.2:c.2179C>T p. (Arg727Cys) and previously published variants NM_001351425.2:c.1997G>A p.(Arg666His)^26^ and c.2588G>C p.(Arg863Pro)^27^ in families 9, 14, and 15; Figures 1 and 3Di, Table 2). All missense variants affect stringently conserved arginine residues (Supplemental Figure 1C) within the IP4P domain surrounding the catalytic CX_5_R motif region (Figures 1B and 3A and D). All are predicted to be deleterious and protein modeling studies identify clear differences in polar contacts (Figure 3Dii-iii), although p.(Arg666His) and p.(Arg863Pro) remain classified as VUS according to ACMG/ACGS criteria (Table 2). The novel NM_001351425.2:c.2179C>T p.(Arg727Cys) variant was further investigated with functional studies described below leading to variant classification as likely pathogenic (Table 2). All variants cosegregated as expected for an autosomal recessive disorder (Figure 1A). Supplemental Table 4 lists other rare variants identified in each family.

### Studies of INPP4A p.(Arg727Cys) fibroblasts from family 9

Because PI(3,4)P_2_ is a critical regulator of Akt-mTOR signal transduction and plays a role in clathrin-mediated endocytosis, we addressed whether the *INPP4A* NM_0013 51425.2:c.2179C>T p.(Arg727Cys) variant leads to defects in these pathways because patient fibroblasts were available for analysis (individual IV:2, family 9, homozygous for INPP4A NM_001351425.2:c.2179C>T p.(Arg727Cys)).

### Endocytic trafficking is impaired in homozygous INPP4A p.(Arg727Cys) fibroblasts and in U2OS cells following knockdown of INPP4A

Transferrin uptake assays are frequently used to study endocytosis and endocytic trafficking. Transferrin receptors bind to extracellular iron-loaded transferrin at the plasma membrane, are delivered to the early endosome via clathrin and dynamin-mediated endocytosis, and subsequently recycled back to the plasma membrane.^40^ CF568-conjugated transferrin uptake can therefore be used to investigate endocytic trafficking by fluorescent microscopy. Accordingly, transferrin uptake into fibroblasts can be blocked by the dynamin inhibitor Dynasore (Figure 3E) and clathrin inhibitor Pitstop (Supplemental Figure 3A). In *INPP4A* NM_001351425.2:c.2179C>T p.(Arg727Cys) fibroblasts, a transferrin uptake assay displayed significantly impaired endocytic trafficking when compared with the unaffected heterozygous mother (III:1, family 9) (Figure 3F). Unaffected transferrin surface levels in *INPP4A* p.(Arg727Cys) fibroblasts (Supplemental Figure 3B) suggest that a combination of receptor endocytosis and receptor recycling to the plasma membrane likely account for impaired transferrin uptake. Knockdown of INPP4A using small interfering RNA in U2OS cells confirmed an endocytic trafficking defect with LoF of INPP4A (Figure 3G and H).

### Downstream Akt and mTORC1 signaling remains intact

Further studies showed that downstream PI(3,4)P_2_-dependent Akt and mTORC1 signaling pathways are largely unaffected, irrespective of whether Akt-mTOR signaling was triggered by refeeding with growth medium or insulin to specifically activate PI3K or amino acids to specifically activate mTORC1 (Supplemental Text 1, Supplemental Figure 3C and D). INPP4A knockdown with small interfering RNA in U2OS cells confirmed the absence of signaling defects upon INPP4A LoF (Supplemental Figure 3E).

## Discussion

Here, we describe extensive genetic, clinical, and molecular investigations in 10 newly identified families, in which our studies identify biallelic *INPP4A* variants as the cause of a severe neurodevelopmental disorder. Concurrently, we reexamined previously published families in which *INPP4A* gene variants were identified as the candidate cause of disease, enabling a significant expansion and more comprehensive description of the clinical spectrum of *INPP4A*-related neurodevelopmental disorder (Figure 1, Table 1, Supplemental Table 1). The cardinal clinical features of this disorder comprise global developmental delay with severe gross motor delay (92% are nonambulatory) and profound speech impairment, severe to profound intellectual disability, and severe lower limb weakness/paralysis (many achieve mobility through crawling or quadrupedal walking). More variable clinical features include cerebellar signs, involuntary movements, axial hypotonia, spasticity, quadriparesis, joint contractures, seizures (myoclonic, GTC, and tonic), visual impairment, and strabismus (Table 1). Neuroimaging findings vary from normal to features of (ponto) cerebellar hypoplasia, ventriculomegaly, reduced cerebral volume and hypomyelination. Short stature (−2.44 to −8.59 SDS below the mean) is a variable and previously unrecognized feature of the condition, identified in 6 (75%) affected individuals (Supplemental Table 1). Microcephaly (−2.54 to −9.77 SDS) is also a prominent feature being present in 15 of 24 individuals. Other previously unreported features include hypodontia and absent central incisors, behavior disorders including agitation/irritability, aggressive episodes, impaired social interaction, and movement disorders, including choreoathetosis, dystonia, and myoclonic jerks.

Loss of INPP4A function likely represents the pathomolecular basis of *INPP4A*-related disorder, supported by the significant constraint of INPP4A (loss-of-function observed/expected upper bound fraction score 0.41; gnomAD v2.1.1)^30^ in genome variant databases. *INPP4A* has numerous transcripts, the matched annotation from National Center for Biotechnology Information and EMBL-EBI (MANE) select transcript (NM_001134225.2) has low expression in brain. Thus NM_001351425.2 was selected as the most representative for brain expression and therefore of most relevance to the condition (Supplemental Figure 2B and C). This was queried by the authors leading to reassessment, consequently NM_001351425.2 has now been defined as the MANE Plus Clinical transcript and will be publicly available in MANE v1.3, reducing the potential for misdiagnosis. Our studies describe 17 families with a total of 11 nonsense or frameshift and 3 missense INPP4A variants. This includes previously published family 17^28^ with a homozygous truncating *INPP4A* c.2726del p.(Gly909Glufs*12) variant located in the last exon, in whom the affected individual displays clinical features, which closely overlap families with pathogenic LoF *INPP4A* variants (Table 1). This truncating variant is predicted to escape NMD (Figure 1B), with the resulting INPP4A protein shown to display aberrant subcellular localization in HeLa cell studies, and is classified as likely pathogenic (Table 2).

Notably, biallelic missense *INPP4A* variants identified in 3 families all cluster in the highly conserved C-terminal IP4P domain (Figures 1B and 3D, Table 2). Our protein modeling studies show that these variants are located in close proximity to the catalytic CX_2_R binding site and that variants p.(Arg727Cys) and p.(Arg863Pro) likely disrupt polar bonds. All affected individuals in these families display significant phenotypic overlap with affected individuals with biallelic LoF *INPP4A* variants (Supplemental Table 1), including features of pontocerebellar hypoplasia on MRI brain imaging. Although 2 previously published missense variants (p.(Arg666His) and p.(Arg863Pro)) remain classified as VUS, our transferrin studies in fibroblasts from affected individual IV:2 from family 9 with a biallelic c.2179C>T p.(Arg727Cys) variant show disruption of endocytic pathways, aligning with the anticipated outcomes of deleterious *INPP4A* variants and supporting the classification of this variant as likely pathogenic (Table 2). Families 10 and 16^25^ in which homozygous *INPP4A* c.2740C>T p.(Arg914*) and c.2744del p.(Asp915Alafs*2) variants were identified are located in intronic regions in all transcript isoforms except NM_001566.2, in which they reside in the last exon (26) and thus are predicted to escape NMD (Supplemental Figure 2B). Although this transcript is considered to be expressed in the brain, the level of expression appears to be lower than the predominant brain transcript (NM_001351425.2; Supplemental Figure 2A and C). These transcript differences provide a potential explanation for the consistent but milder phenotypes observed in these families. However, because further conclusive functional evidence is required to confirm this hypothesis, and the clinical relevance of these variants remains unclear, they are classified as VUS.

Twelve affected individuals (families 1-3 and 11) with *INPP4A*-related disorder associated with homozygous nonsense variants in exon 4 of *INPP4A* (c.115C>T p.(Gln39*) and c.137T>G p.(Leu46*)) appear to lack features of spasticity, limb hypertonia, and hyperreflexia. Additionally, seizure presentations appear to be less severe in these families with affected individuals displaying no seizures or limited to tonic seizures, which are well controlled with risperidone and a ketogenic diet (II:1, family 2), or febrile seizures that resolved after infancy with no medication (IV:2, family 11). Neuroimaging was reported as unremarkable in 6 of these individuals (unavailable for review in 5 cases), including MRI neuroimaging for individual II:1 (family 2; Figure 2iiA) shows normal appearance, with no evidence of (ponto)cerebellar hypoplasia or other brain abnormalities. Five of these individuals were reported to have additional dental anomalies, including hypodontia and absent central incisors (Figure 2i). This contrasts significantly with 9 affected individuals (families 4-8 and 12-13) with homozygous LoF *INPP4A* variants located downstream of exon 4 all of whom display severe seizure phenotypes, including myoclonic, GTC, and tonic seizures, with onset between the neonatal period to 18 months of age. Seizures were intractable in 6 affected individuals, leading to death from status epilepticus in individual II:1, family 17 at the age of 27 months. Other consistent features in these individuals include spasticity or hypertonia/hyperreflexia, paraparesis/paraplegia, and microcephaly, with significant anomalies on MRI brain imaging; all have cerebellar hypoplasia (6 with pontocerebellar hypoplasia), with variable features of ventriculomegaly, reduced cerebral volume, thinning of the corpus callosum, and hypomyelination. Five of these individuals also have joint contractures, 3 had a gastrostomy, and 2 required tracheostomies.

Interestingly, these striking genotype-phenotype differences are also mirrored in Inpp4a^−/−^ mouse models. Three mouse models have been described including the *weeble* mouse with a spontaneous homozygous frameshift (c.744delG p.(Gln248Gln*15)) LoF *Inpp4a* variant^21^ and two *Inpp4a*^−/−^ knockout mouse strains targeting exons 3-4^19,41^ and exon 25^41^ (previously described in terms of coding exons 1-2, and exon 23, Supplemental Figure 2D). All mouse models display a phenotype mirroring human *INPP4A*-related neurodevelopmental disorder entailing a severe movement disorder with inability to walk, a small brain, and poor growth/weight gain. However, there are phenotypical differences between these models, with the exon 25 *Inpp4a*^−/−^ and *weeble* mice being ataxic and the exon 3-4 *Inpp4a*^−/−^ mouse displaying an involuntary movement disorder, including limb hyperkinesias (choreas, ballism), opisthotonus, and dystonia. Similarly, the exon 3-4 *Inpp4a*^−/−^ and *weeble* mice show seizure activity, whereas the exon 25 *Inpp4a*^−/−^ mice do not.^21,41^ There are also differences in the neuroanatomical defects present in each mouse consistent with their differing motor phenotypes; histological examination of the exon 3-4 *Inpp4a*^−/−^ mouse identified mild degeneration of the striatum with mediumsized spiny projection neurons particularly affected but no cerebellar hypoplasia or other brain abnormalities. This is comparable to the normal neuroimaging findings observed in individual II:1 in family 2 (Figure 2iiA) with no cerebellar hypoplasia detected in any affected individual homozygous for LoF *INPP4A* variants in exon 4 (Table 1). In contrast, the exon 25 *Inpp4a*^−/−^ and *weeble* mice display severe neuronal loss in the cerebellum with pronounced Purkinje and pyramidal cell degeneration associated with cell death during postnatal development and milder cell loss generally within the cerebral cortex.^21,41^ This is strikingly similar to neuroanatomical findings in all affected individuals with homozygous *INPP4A* LoF variants downstream of exon 4, with severe cerebellar hypoplasia and reduced cerebral volume (Figure 2iiB-L). These findings further support a distinct phenotypic presentation in affected individuals with biallelic LoF *INPP4A* variants affecting exon 4 compared with affected individuals with biallelic downstream *INPP4A* LoF variants. The phenotypic differences in the mice have been postulated to be caused by retained phosphatase activity in N-terminal truncated INPP4A protein.^41^ Ectopic expression of INPP4A-Δexon3,4-FLAG protein from constructs transfected into HEK293T cells showed no significant reduction in Inpp4a phosphatase activity compared with wild type, although expression of INPP4A-Δexon25-FLAG protein showed a loss of phosphatase activity.^41^ Studies of exon 3-4 *Inpp4a*^−/−^ mice also confirm expression of both INPP4A isoforms alpha and beta in the mouse cerebellum compared with other brain regions, potentially explaining the prominent cerebellar manifestations of *INPP4A*-related disorder.^41^ Further, this potentially also explains the milder phenotype of affected individuals from families 10 and 16 with homozygous *INPP4A* variants only affecting the NM_001566.2 transcript. This transcript corresponds to the cerebellum-specific Inpp4a beta isoform in mice and provides a plausible explanation for the predominant cerebellar involvement observed in individual II:1 from family 10 (see Supplemental Video 2). Exon 4 is integral to all identified INPP4A transcript isoforms (Supplemental Figure 2B); therefore, the observed genotype-phenotype correlation is unlikely to be explained by expression of transcript isoforms unaffected by the exon 4 variants. We thus explored whether translation reinitiation at start sites located downstream of exon 4 may explain these phenotypical differences, through leaky expression because reinitiation can occur when the distance between the canonical start and the stop codon is short, within the first ~70 amino acids,^42^ which increases the probability that initiation factors are still present. This identified several candidate downstream in-frame noncanonical initiation sites shared by both mice and humans, potentially generating an N-terminal truncated INPP4A protein (Supplemental Figure 2E, Supplemental Table 2). The first of these sites of moderate Kozak strength would be expected to generate a polypeptide lacking the 54 N-terminal amino acids including the first beta-strand of the C2A domain (Figure 3C), which may retain some molecular function that could lead to sparing of structural brain abnormalities and associated clinical features in affected individuals. Supportive evidence for alternative downstream initiation was identified in long read RNA-seq transcript data from 9 healthy adult dorsolateral prefrontal cortex samples identifying 34 novel INPP4A protein coding brain transcripts, downstream of the exon 4 nonsense variants (Supplemental Table 5). The C2A domains bind PtdIns(3,4) P_2_ and PtdIns(4)P, potentially involved in subcellular targeting of INPP4A to membranes enriched in these phosphoinositides.^15,43^ This is compatible with the aggregate-like structures seen in the cytoplasm in subcellular localization studies of NIH3T3 cells transiently transfected with INPP4A-Δexon3,4-FLAG constructs,^41^ indicative of a role of the N terminus C2 domain in subcellular localization. Further studies are required to elucidate the underlying pathomechanism of these apparent phenotypic differences in both humans and mice (Supplemental Table 3).

INPP4A catalyzes the dephosphorylation of PtdIns(3,4) P_2_ to PtdIns(3)P; therefore, absence of INPP4A leads to delayed degradation of PtdIns(3,4)P2 and increased downstream PI3K-AKT signalling^15,16^ (Supplemental Figure 1A and B). Individuals with INPP4A-related disorder have microcephaly and do not display features consistent with overgrowth observed in mTORopathies.^44^ Consistent with this, our functional studies of fibroblasts from individual IV:2 from family 9 (c.2179C>T p.(Arg727Cys)) show that PI(3,4)P_2_-dependent Akt and mTORC1 signaling pathways are largely unaffected (Supplemental Figure 3C and D) and are unlikely to represent the pathomechanism of INPP4A-related disorder. Our preliminary fibroblast cell studies further identify disruption of endocytic pathways (Figure 3E and F) as the likely mechanism of disease, consistent with previous findings of a role of INPP4A in endocytosis,^13,14^ and highlight the value of further studies in this area in samples from larger numbers of affected individuals to more precisely characterize how INPP4A variants specifically impair endocytic processes.

INPP4A has also been shown to play a protective role against NMDA receptor-mediated excitotoxic death of neurons, through regulation of NMDAR localization at the postsynaptic density,^19^ possibly mediated by an effect on endocytosis.^45^ Lack of INPP4A expression in Inpp4a^−/−^ mouse striatal cells leads to an increase in postsynaptic NMDARs with larger NMDA-mediated currents observed, and potentiated glutamate-induced striatal neuron cell death, with toxic effects of glutamate at lower concentrations. Further in vitro studies confirm that knockdown of INPP4A in an epilepsy cell model results in increased apoptosis through increased calcium release.^18^ This may potentially explain the increased susceptibility to seizure development and neurodegeneration in both weeble and exon 3–4 Inpp4a^−/−^ mice and individuals with *INPP4A*-related disorder. Further studies have identified that expression of a specific glutamate transporter (SLC1A6) may have a protective effect on Purkinje neuronal cell loss observed with absence of INPP4A in weeble mice.^46^ Expression levels of other proteins affecting neuronal excitotoxicity in different regions of the brain may also explain the severity and variability of features observed in individuals with INPP4A-related disorder. Importantly, previous studies in Inpp4a^−/−^ mice show that NMDAR antagonist MK-801 improves hindlimb clasping and reduces seizures,^19^ suggesting that pharmacotherapeutic developments may well be possible for treatment of individuals affected by INPP4A-related neurodevelopmental disorder, providing a potential avenue to future research studies.

Here, we more precisely define the clinical phenotype, and genetic/molecular basis, of INPP4A-related neurodevelopmental disorder. Our findings have enabled us to propose genotype-specific guidelines to aid the clinical management and genetic counseling of individuals affected by INPP4A-related disorder and their parents (summarized in Supplemental Table 6), targeted at preventing lifethreatening complications, including monitoring for feeding problems in the neonatal period/early infancy and early identification and optimization of antiepileptic medications to maintain seizure control, as well as optimizing psychomotor development and functional level.

## Supplementary Material

SupplementalTables

SupplementaryMaterial

SupplVideo1

SupplVideo4

SupplVideo2

## Figures and Tables

**Table 1:** A comparison of clinical features of 26 affected individuals with confirmed and suspected *INPP4A*-related neurodevelopmental disorder

Clinical Feature	Exon 4 nonsense (12 (2))	Other LoF (9)	Missense (5 (3))	Total cases (26 (5))
Sex	F = 5(2), M = 7	F = 3, M = 6	F = 3(2), M = 2(1)	F = 11(4), M = 15(1)
Mean age in years (range)	25.5 (4 – 40)	6.8 (0.5 – 27)	7.2 (1-13)	15.5 (0.5 – 40)
**Developmental phenotype**				
Intellectual disability	100% (12(2)/12)	100% (9/9)	100% (5(3)/5)	100% (26(5)/26)
- Moderate	8% (1/12)	0% (0/8)	20% (1/5)	8% (2/25)
- Severe	92% (11(2)/12)	37% (3/8)	40% (2(1)/5)	64% (16(3)/25)
- Profound	0% (0/12)	63% (5/8)	40% (2(2)/5)	28% (7(2)/25)
Global developmental delay	100% (12(2)/12)	100% (9/9)	100% (5(3)/5)	100% (26(5)/26)
- Severe speech delay (few sounds)	92% (11(2)/12)	100% (8/8)	100% (5(3)/5)	96% (24(5)/25)
- Non-verbal	8% (1(1)/12)	100% (8/8)	40% (2(2)/5)	44% (11(3)/25)
- Non-ambulatory (>1.5 years)	92% (11(1)/12)	100% (8/8)	75% (3(3)/4)	92% (22(4)/24)
- Unable to sit unsupported	8% (1(1)/12)	100% (8/8)	50% (2(2)/4)	46% (11(3)/24)
**Neurological features**				
Microcephaly (≤ −2SDS)	30% (3(2)/10)	100% (9/9)	60% (3(3)/5)	63% (15(5)/24)
Cerebellar signs	40% (2(2)/5)	100% (6/6)	100% (5(3)/5)	81% (13(5)/16)
Axial hypotonia	22% (2/9)	86% (6/7)	50% (2(2)/4)	50% (10(2)/20)
Paraparesis/paraplegia	100% (12(2)/12)	100% (7/7)	60% (3(3)/5)	92% (22(5)/24)
Quadriparesis	67% (8(2)/12)	100% (7/7)	60% (3(3)/5)	75% (18(5)/24)
Spasticity	0% (0/12)	75% (6/8)	60% (3(3)/5)	36% (9(3)/25)
Hyperreflexia	0% (0/12)	75% (6/8)	80% (4(2)/5)	44% (11(2)/25)
Dystonia/choreoathetosis/myoclonus	25% (3(1)/12)	57% (4/7)	0% (0/5)	29% (7(1)/24)
Seizures	8% (1/12)	100% (8/8)	60% (3(3)/5)	48% (12(3)/25)
- Severe, intractable	0% (0/1)	75% (6/8)	67% (2(2)/3)	67% (8(2)/12)
- Myoclonic	0% (0/1)	63% (5/8)	67% (2(2)/3)	58% (7(2)/12)
- Generalised tonic-clonic	0% (0/1)	75% (6/8)	100% (3(3)/3)	67% (8(3)/12)
- Tonic	100% (1/1)	50% (4/8)	33% (1(1)/3)	50% (6(1)/12)
Neuroimaging	50% (6/12)	100% (9/9)	100% (5(3)/5)	77% (20(3)/26)
- Normal	100% (6/6)	0% (0/9)	0% (0/5)	30% (6/20)
- Cerebellar hypoplasia	0% (0/6)	100% (9/9)	80% (4(2)/5)	65% (13(2)/20)
- Hindbrain abnormality	0% (0/6)	67% (6/9)	40% (2(2)/5)	40% (8(2)/20)
- Reduced cerebral volume	0% (0/6)	56% (5/9)	60% (3(3)/5)	40% (8(3)/20)
- Ventriculomegaly	0% (0/6)	44% (4/9)	0% (0/5)	20% (4/20)
- Thinning of the corpus callosum	0% (0/6)	22% (2/9)	40% (2/5)	20% (4/20)
- Hypomyelination	0% (0/6)	22% (2/9)	20% (1(1)/5)	15% (3(1)/20)
**Behavioural phenotype**				
Agitation/irritability	0% (0/6)	67% (4/6)	0% (0/4)	25% (4/16)
Withdrawn/poor social interaction	50% (3(2)/6)	17% (1/6)	40% (2/5)	35% (6(2)/17)
**Vision**				
Strabismus	42% (5(2)/12)	25% (2/8)	20% (1/5)	32% (8(2)/25)
Reduced visual acuity/cortical blindness	0% (0/12)	88% (7/8)	60% (3(3)/5)	40% (10(3)/25)
**Other features**				
Short stature (≤ −2SDS)	100% (3(2)/3)	60% (3/5)	25% (1/4)	58% (7(2)/12)
Feeding problems (including gastrostomy)	33% (1/3)	44% (4/9)	20% (1(1)/5)	35% (6(1)/17)
Joint contractures/arthrogryposis	8% (1/12)	63% (5/8)	0% (0/5)	24% (6/25)
Hypodontia	42% (5(2)/12)	0% (0/8)	0% (0/5)	20% (5(2)/25)

Subdivided into clinical features of 12 affected individuals homozygous for exon 4 *INPP4A* nonsense variants, 9 individuals homozygous for other *INPP4A* variants downstream of exon 4, and 5 individuals homozygous for *INPP4A* missense variants. Percentage and proportion (brackets) of affected individuals with each clinical feature is shown. Gray text indicates affected individuals with biallelic *INPP4A* VUS.

*F*, female; *LoF*, loss of function; *M*, male; *SDS*, standard deviation score.

Height and head circumference *z*-scores were calculated using a Microsoft Excel add-in to access growth references based on the LMS method^32^ using UK 1990 reference population.^33^

**Table 2:** Homozygous *INPP4A* (NM_001351425.2) variants identified in affected individuals from 17 families with confirmed or suspected *INPP4A*-related disorder

CASES	INPP4A NM_001351425.2	GRCh38 (hg38):	NMD predicted?	gnomAD v2.1.1 HET	gnomAD v4.1.0 HET	gnomAD HOM	gnomAD All AF	SIFT	Polyphen-2	REVEL	Splice AI	ACMG/ACGS Class
**Published here:**												
Family 3	c.137T>G p.(Leu46*)	NC_000002.12: g.98520717T>G	N	0	0	0	0	NA				**VUS** PVS1 (moderate), PM2, PM3 (supporting)
Family 4	c.322_323del p.(Leu108Tyrfs*13)	NC_000002.12: g.98535776_98535777del	Y	0	0	0	0	NA				**Pathogenic** PVS1, PM2, PM3 (supporting)
Families 5 and 6	c.352_353del p.(Ser118Argfs*3)	NC_000002.12: g.98535807_98535808del	Y	0	0	0	0	NA				**Pathogenic** PVS1, PM2, PM3
Family 7	c.981del p.(Asp328Ilefs*4)	NC_000002.12: g.98546000del	Y	0	0	0	0	NA				**Pathogenic** PVS1, PM2, PM3 (supporting)
Family 8	c.1764G>A p.(Trp588*)	NC_000002.12: g.98563472G>A	Y	0	1	0	0.0000006848	NA				**Pathogenic** PVS1, PM2, PM3 (supporting)
Family 9	c.2179C>T p.(Arg727Cys)	NC_000002.12: g.98565765C>T	N	1	1	0	0.0000006853	0.001	0.999	0.662	0.02	**Likely pathogenic** PS3 (moderate), PM1 (supporting), PM2, PM3 (supporting)
Family 10	**NM_001566.2** c.2740C>T p.(Arg914*)	NC_000002.12: g.98581631C>T	N NM_001566.2 only	1	8	0	0.000005076	NA				**VUS** PM2, PM3 (supporting)
**Previously published:**												
Families 1, 2 and 11 Banihashemi et al^23^	c.115C>T p.(Gln39*)	NC_000002.12: g.98520695C>T	N	0	2	0	0.000001489	NA				**Pathogenic** PVS1 (moderate), PM2, PM3, PP1 (strong)
Family 12 Özkan Kart et al^24^	c.646C>T p.(Arg216*)	NC_000002.12: g.98538957C>T	Y	0	1	0	0.0000006864	NA				**Pathogenic** PVS1, PM2, PM3 (supporting)
Family 13 Sheffer et al^22^	c.1585-81_1723+ 1551del p.? (1770bp deletion)	NC_000002.12: g.98555472_98557243del	Y	0	0	0	0	NA				**Pathogenic** PVS1, PM2, PM3 (supporting)
Family 14 Olson et al^26^	C.1997G>A p.(Arg666His)	NC_000002.12: g.98564707G>A	N	7	50	0	0.00003116	0.072	0.904	0.209	0.00	**VUS** PM1 (supporting), PM2, PM3 (supporting)
Family 15 Al-Kasbi et al^27^	c.2588G>C p.(Arg863Pro)	NC_000002.12: g.98577044G>C	N	0	0	0	0	0.000	0.999	0.797	0.00	**VUS** PM1 (supporting), PM2, PM3 (supporting), PP3
Family 16 Najmabadi et al^25^	**NM_001566.2** c.2744del p.(Asp915Alafs*2)	NC_000002.12: g.98581635del	N NM_001566.2 only	0	0	0	0	NA				**VUS** PS3 (supporting), PM2, PM3 (supporting), PP1 (moderate)
Family 17 Hecher et al^28^	c.2726del p.(Gly909Glufs*12)	NC_000002.12: g.98587513del	N	0	0	0	0	NA				**Likely pathogenic** PVS1 (moderate), PS3 (supporting), PM2, PM3 (supporting)

*ACGS*, Association for Clinical Genomic Science^35^; *ACMG*, American College of Medical Genetics^34^; *AF*, allele frequency; *gnomAD*, Genome Aggregation Database; *HET*, heterozygous; *HOM*, homozygous; *NA*, not applicable; *NK*, not known; *N*, no; *NMD*, nonsense-mediated decay; *Polyphen-2*, polymorphism phenotyping2^2,37^; *REVEL*, rare exome variant ensemble learner^38^; *SIFT*, sorting intolerant from tolerant^36^; *splice AI*, splice artificial intelligence^39^; *VUS*, variant of uncertain significance; *Y*, yes.

## Data Availability

Data supporting this work are available upon request. Clinical data sets have been deidentified.
